# Autistic traits in children with ADHD index clinical and cognitive problems

**DOI:** 10.1007/s00787-013-0398-6

**Published:** 2013-04-25

**Authors:** Miriam Cooper, Joanna Martin, Kate Langley, Marian Hamshere, Anita Thapar

**Affiliations:** Child and Adolescent Psychiatry Section, Institute of Psychological Medicine and Clinical Neurosciences, MRC Centre for Neuropsychiatric Genetics and Genomics, Cardiff University School of Medicine, Heath Park, Cardiff, CF14 4XN UK

**Keywords:** ADHD, ASD, Comorbidity, Cognitive deficits, Neurodevelopment

## Abstract

Traits of autistic spectrum disorders (ASD) occur frequently in attention deficit hyperactivity disorder (ADHD), but the significance of their presence in terms of phenotype and underlying neurobiology is not properly understood. This analysis aimed to determine whether higher levels of autistic traits, as measured by the Social Communication Questionnaire (SCQ), index a more severe presentation in a large, rigorously phenotyped sample of children with ADHD (*N* = 711). Regression analyses were used to examine association of SCQ scores with core ADHD features, clinical comorbidities and cognitive and developmental features, with adjustment for putative confounders. For outcomes showing association with total SCQ score, secondary analyses determined levels of differential association of the three ASD sub-domains. Results suggest that increasing ASD symptomatology within ADHD is associated with a more severe phenotype in terms of oppositional, conduct and anxiety symptoms, lower full-scale IQ, working memory deficits and general motor problems. These associations persisted after accounting for ADHD severity, suggesting that autistic symptomatology independently indexes the severity of comorbid impairments in the context of ADHD. Sub-domain scores did not show unique contributions to most outcomes, except that social deficits were independently associated with oppositional symptoms and repetitive behaviours independently predicted hyperactive-impulsive symptoms and motor problems. It would be worthwhile for clinicians to consider levels of socio-communicative and repetitive traits in those with ADHD who do not meet diagnostic criteria for ASD, as they index higher levels of phenotypic complexity, which may have implications for efficacy of interventions.

**Electronic supplementary material:**

The online version of this article (doi:10.1007/s00787-013-0398-6) contains supplementary material, which is available to authorized users.

## Introduction

Autistic spectrum disorders (ASD) and attention deficit hyperactivity disorder (ADHD) are childhood-onset neurodevelopmental disorders. Although there are some important differences (e.g., core symptom definition and recommended treatment), ASD and ADHD share many similar impairments in developmental and cognitive domains. Both are substantially more common in boys than girls, with a gender ratio of around 6:1 [1, 2]. There is strong comorbidity of both disorders with intellectual disability (ID) [3–6]. Both are also associated with other specific learning and developmental problems, notably reading difficulties [7–9] and motor problems, including developmental co-ordination disorder [10, 11]. Specific speech and language problems similar to those in ASD are frequently seen in those with ADHD [12], and general language delay, one of the defining features of ASD, is also commonly seen in ADHD [13]. Furthermore, both disorders are associated with deficits in executive functions (EF) [14, 15].

Importantly, ASD and ADHD also show remarkably high comorbidity with each other, at trait and full disorder level, both in clinical [16–19] and general population [20–23] cross-sectional samples. Further support for this comes from a recent longitudinal population twin study, which showed that traits of one condition at age 8 are significantly associated with traits of the other condition at age 12, with a stronger association for ADHD predicting ASD than vice versa [24]. Furthermore, whilst the neurobiology of both disorders is yet to be fully elucidated, there is evidence from genetic [25] and imaging studies [26] that they may share underlying neurobiological dysfunction.

Current diagnostic classification systems (DSM-IV and ICD-10) preclude the co-diagnosis of ADHD in children with ASD. However, with comorbidity estimates being so high, the DSM-5 will no longer have this restriction [27]. In view of these potential changes and research highlighting the overlap across these two conditions, there have been a number of attempts to shed light on the similarities between children with a primary diagnosis of ADHD or ASD and children meeting diagnostic criteria or having a high level of traits of both conditions, in terms of their core neurodevelopmental phenotypes, comorbid problems and cognitive and developmental profiles. For the purpose of this study, we focus on the presence of elevated ASD traits in children with ADHD.

### Core ADHD symptom profile of ADHD children with elevated ASD traits

The profile of ADHD symptoms in children with a primary diagnosis of ADHD does not appear to be influenced by the presence of high ASD traits [28, 29]. However, high ASD traits may be associated with a greater likelihood of a combined subtype of ADHD [29]. Others have shown that ASD traits assessed continuously within a clinical ADHD sample are predicted by higher levels of hyperactive-impulsive symptoms but not inattentive symptoms [30], although there was a trend towards association with inattention in the study. It is important to note that these studies generally screen for and exclude children with a diagnosis of ASD. To date, results are conflicting and it is not clear whether the presence of autistic traits in children diagnosed with ADHD indexes a typical, or indeed more severe, presentation in terms of the core ADHD symptoms and diagnosis subtype. Further studies are necessary to clarify the inconclusive findings.

### Comorbidity in ADHD children with elevated ASD traits

Research on comorbid psychopathology differences in children with ADHD with high vs. low levels of ASD traits is also mixed. One study found no clear differences [29], whereas another study, which utilised latent class analysis in a sample of children with ADHD to distinguish clusters based on ASD traits, found higher rates of comorbid oppositional defiant disorder (ODD) and conduct disorder (CD) in children with high ASD symptoms [31]. Others have assessed ASD and ADHD traits continuously within a clinical ADHD sample and found an association between high ASD trait scores and presence of maternal ASD traits, but did not find an association with ODD, CD or anxiety symptoms [30]. Taken as a whole, the evidence is limited and mixed and it is not clear whether children with a diagnosis of ADHD who have traits of ASD are more likely to show elevated comorbid problems.

### Cognitive/developmental profiles of ADHD children with elevated ASD traits

In terms of general cognitive ability, the presence of ASD traits in children with ADHD does not appear to be associated with IQ [29, 32]. However, studies generally exclude children with IQ < 70 and, therefore, the full extent of impairments in IQ in ADHD children with elevated ASD traits has not been fully explored. A recent review of the few studies examining deficits in various EF sub-domains simultaneously in children with ADHD and ASD suggests that EF deficits appear qualitatively similar in both conditions but can vary in degree of severity [33]. However, this review also highlights the lack of sufficient research directly comparing children with traits of both phenotypes relative to those with only one phenotype; studies to date are small, with inconsistencies in methodology, inclusion criteria and the specific EF sub-domains tested, making it difficult to make any firm conclusions. In terms of other developmental domains, there is some evidence that autistic traits in children with ADHD are associated with higher rates of motor and language problems [31]. Further studies are necessary to confirm these findings and clarify the specifics of the relationship between ASD traits and cognitive and developmental deficits in children with ADHD.

### Rationale for current study

Although in summary, there are a number of studies beginning to address whether autistic traits in children with ADHD index a more severe clinical, cognitive and developmental profile [28–32], interpretation of findings is complicated by methodological heterogeneity between studies. We aim to clarify and extend previous research using a large, rigorously phenotyped ADHD sample (with no known clinical diagnosis of and, therefore, unselected for ASD), assessing ASD traits using a continuous scale rather than a cut-off point, addressing putative confounders (age, gender, IQ, socioeconomic status) and including children across the full range of intellectual abilities.

Based on previous literature, we hypothesise that the presence of an increasing number of ASD symptoms in those with ADHD may be associated with combined subtype ADHD diagnosis, a greater number of ADHD symptoms, more severe comorbid anxiety, depression, ODD and CD problems, lower IQ, lower reading ability, more EF problems and a greater rate of speech and motor problems. Although traditionally regarded as clustering very strongly, the three domains of ASD (deficits in social interaction, communication problems and restrictive/repetitive behaviours) have been suggested to dissociate, both in terms of clinical presentation and in terms of underlying genetic aetiology [34]. As a secondary analysis, we thus seek to determine whether these three sub-domains of ASD symptoms are differentially associated with any of the outcomes which show an association with total ASD trait scores (i.e., if any sub-domain is independently associated with the outcome over and above the other sub-domains).

## Methods

### Sample

Children with a confirmed or suspected clinical diagnosis of ADHD were recruited through child psychiatry and paediatric outpatient clinics in the United Kingdom for a study of genetic and environmental influences on ADHD. Approval for the study was obtained from the North West England and Wales Multicentre Research Ethics Committees and written informed consent to participate was obtained from parents, and assent/consent was gained from the children.

Children were eligible for inclusion in this current analysis if they had a current diagnosis of ADHD DSM-III-R or DSM-IV with complete subtype data available and sufficient ASD trait data for analysis (see “Measures” section below for details). Exclusion criteria were a known clinician’s diagnosis of ASD or schizophrenia (screened for using the referral form), history of epilepsy or other neurological disorder, and diagnosis of bipolar disorder or Tourette’s syndrome (as confirmed by diagnostic interview). Presence of ID was not an exclusion criterion for the current analyses. These criteria resulted in a total of *N* = 711 young people included in the analyses. There were 113 females (15.9 %) in the sample and the children were aged between 5 and 18 years (mean = 10.3, SD = 2.9).

### Measures

Research diagnoses of ADHD and psychiatric comorbidities were ascertained using the parent version of the Child and Adolescent Psychiatric Assessment (CAPA) [35], a semi-structured interview. The CAPA provides both symptom counts and categorical diagnoses of the presence of psychiatric disorders. The comorbid conditions assessed were DSM-IV ODD, CD, anxiety disorders (generalised anxiety disorder, social anxiety and separation anxiety), depression and mania. As a secondary analysis of CD problems, CD symptoms were divided into aggressive and covert symptoms as suggested by previous factor analyses [36]. Comorbid anxiety and depression symptoms were also assessed using the child version of the CAPA [37] for children aged 12 years and over; these were endorsed if either parent or child reported their presence. CAPA interviews were undertaken by trained psychologists and cases were discussed weekly with a child psychiatrist. Inter-rater reliability for ADHD diagnostic subtype was perfect (*κ* = 1.0). Inter-rater reliability for parent-rated CD symptoms was very good (intra class correlation = 0.98). Confirmation of pervasiveness of ADHD symptoms in school was obtained using the Child ADHD Teacher Telephone Interview (CHATTI) [38], the Conners Teacher Rating Scale [39] or the DuPaul rating scale [40]. ADHD diagnosis was considered as a binary outcome, with DSM-III-R-only as well as inattentive and hyperactive-impulsive subtypes collapsed into a single group (*N* = 192) and compared with those with a diagnosis of combined subtype ADHD (*N* = 519).

ASD traits were analysed as a continuous measure using the Social Communication Questionnaire (SCQ), (formerly known as the Autism Screening Questionnaire, ASQ) [41, 42]. The SCQ is a 40-item parent-rated questionnaire, based on the Autism Diagnostic Interview-Revised (ADI-R) [43]. These trait scores were examined in a continuous manner as there is evidence that autistic traits are continuously distributed throughout the population [44]. All analyses are conducted on SCQ total scores in children who have ADHD but no known clinician’s diagnosis of an ASD. Item 1 is a language screening item and was omitted from the total score. For consistency and comparability, where parents answered ‘no’ to this item and consequently omitted the following seven questions, these children were not included in analyses (*N* = 20). The remaining 39 items were divided into the three sub-domains of autistic symptoms, as defined by the diagnostic symptoms stipulated by the DSM-IV and ICD-10; there were 20 items classed as ‘social deficits’, 10 as ‘communication deficits’ and 8 as ‘restricted and repetitive behaviours (RRBs)’. Totals for each sub-domain were calculated based on these items. Item 18 was unclassified as it is not a part of the diagnostic criteria for ASD. Details of item classification can be found in Supplementary Table 1. To account for missing questionnaire items in calculating the total SCQ score and sub-domains, a prorated score was calculated for children with 10 % or fewer missing items, where the fraction of endorsed items out of those completed was multiplied by the total number of items (39). Children with >10 % missing on any sub-domain or total were omitted from analyses (*N* = 107). Group comparisons showed that these children with excessive missing data had lower IQ (*p* = 0.03) than the children included in the final sample (*N* = 711), although they did not differ in terms of gender, age at assessment, family socioeconomic status (SES) or severity of their ADHD symptoms.

The Wechsler Intelligence Scale for Children (WISC-III and WISC-IV) [45, 46] was used to assess full-scale IQ, using all subtests. The Digit Span subtest is a measure of verbal working memory and has been used in previous research to assess this domain of functioning in children with ADHD [47] and ASD [48]. The Wechsler Objective Reading Dimensions (WORD) [49] was used to assess reading, spelling and reading comprehension abilities in children up to the age of 12. The intra–extra dimensional shift (IED) task from the Cambridge Neuropsychological Battery (CANTAB) [50] was used as a measure of EF (visual discrimination, set shifting and attentional flexibility). The measure used was whether the child had successfully completed stage 8, the crucial stage at which the extra-dimensional shift first occurs; this variable measures ability to shift attention to a previously irrelevant feature (known as set-shifting), akin to a change in category in the Wisconsin Card Sorting Task (WCST) [51] and has been found to be particularly impaired in unmedicated children with ADHD [52, 53] and in children with ASD [54, 55] relative to controls.

All cognitive assessments were performed by trained psychologists. Parents were asked to withhold stimulant medication from their child for 24 h prior to testing. Parents were also asked general questions about their child’s speech and motor development at the interview; these were: “was your child talking by age 2?”, “has your child ever had speech therapy?”, “was your child walking by 18 months?”, and “is your child clumsy?—not at all,—just a little,—pretty much,—very much”. Children who were not talking by age 2 and/or had had speech therapy were classed as positive for this broad measure of language problems. Children who were not walking by 18 months and/or were clumsy “pretty much” or “very much” were classed as positive for this broad measure of motor problems.

Family annual income, parental employment status and parental educational attainment were assessed by parental questionnaire. Low income was defined as annual family income <£20,000 (equivalent ~$32,000), and low educational attainment was defined as having left school without qualifications (GCSE or equivalent) at the age of 16. SES was classified by the occupation of the main family wage earner in the family using the UK Standard Occupation Classification 2000 [56]. Three SES categories were defined (low: unskilled workers/unemployed; medium: manual and non-manual skilled/partially skilled workers; high: professional and managerial workers). Medium and high categories were merged to give binary categories of low SES or otherwise.

### Data analyses

All analyses were performed using SPSS version 18. All tests were two-tailed. Where a variable was not normally distributed, the scores were natural logarithmically transformed (ln *x* + 1), and analyses were run on transformed scores (which were all normally distributed). First, the effect of putative covariates was assessed using simple logistic and linear regressions. Next, logistic and linear regressions were used to determine the associations of total SCQ score (predictor variable) with clinical, cognitive and developmental characteristics (outcomes) of the ADHD sample. Our primary model analysed unadjusted associations between SCQ scores and outcomes in the full sample. Next, we included covariates to determine to what extent observed associations were explained by other variables. IQ, age, gender and family SES were included as covariates; children with any one of these covariates missing (*N* = 112) could not be included in this model. Finally, severity of ADHD symptoms was added as a further covariate to determine whether ADHD symptom levels contributed to the observed associations. However, IQ was not included as a covariate in the analysis of the WISC-IV Digit Span subtest in view of it being a component of full-scale IQ. Owing to the number of primary tests performed, correction for multiple testing was administered using Bonferroni; alpha was set at *p* = 0.003 (0.05/19) for 19 tests performed (11 tests of clinical outcomes, 6 tests of cognitive outcomes and 2 tests of developmental outcomes).

As a secondary test of CD problems, aggressive and covert CD symptoms were entered into a multivariate regression model together, first unadjusted and then after adjusting for all covariates. This was to test whether either type of CD symptoms independently contributed to any observed association between the CD symptoms and total SCQ score.

As a secondary test of IQ abilities, the four WISC-IV indices were entered simultaneously into one multivariate regression model, to determine the level of each index’s unique contribution to variance in SCQ scores (i.e., to determine whether any of the indices are independently associated with the outcome over and above the other indices). Given that these indices are components of IQ, this multivariate model did not include IQ as a covariate.

For outcome variables that showed association with overall SCQ score, secondary analyses were performed on the three SCQ sub-domain scores (social deficits, communication deficits and RRBs), to see whether scores on any of the sub-domains predicted a greater association with the outcome variables above and beyond shared variance with the other sub-domains. For each outcome variable, covariates (age, IQ, gender and family SES) and all three SCQ sub-domain scores were entered into a single multivariate model to test for association with the outcomes. As these sub-domain analyses were exploratory and secondary, the same threshold for multiple testing (*p* < 0.003) was used when interpreting the results.

## Results

**Fig. 1:**
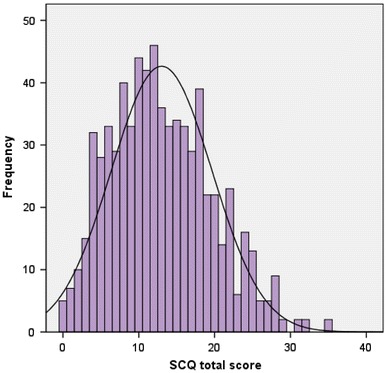
Frequency distribution of total Social Communication Questionnaire score in the sample (*N* = 711)

Table 1 shows general sample characteristics, including mean total SCQ by each of the descriptive and diagnostic variables. Total SCQ scores and IQ scores were normally distributed and IQ ranged between 43 and 124 (mean = 84.3, SD = 14.0), which included 85 children with an IQ < 70. Figure 1 displays the frequency distribution of SCQ scores across the sample. Total SCQ scores were associated with lower full-scale IQ (*B* = −0.45, *p* = 5.7 × 10^−8^), greater rate of low family income [odds ratio (OR) = 1.04, 95 % confidence intervals (CI) = 1.01–1.07, *p* = 0.006] and low family SES (OR = 1.04, 95 % CI = 1.01–1.06, *p* = 0.003). There was no association of SCQ and child’s age at assessment (*B* = −0.02, *p* = 0.31), gender (OR = 1.01, 95 % CI = 0.98–1.04, *p* = 0.54) or parental education (OR = 1.00, 95 % CI = 0.97–1.03, *p* = 0.91).

**Table 1:** Sample characteristics

Variable		*N*	%	SCQ mean (SD)
Gender	Male	598	84.1	13.1 (6.7)
	Female	113	15.9	12.7 (6.3)
Family income	Low income	285	63.3	13.7 (7.2)
	Medium–high income	165	36.7	11.8 (6.6)
Parental education	No GCSEs	128	27.1	13.0 (6.9)
	GCSEs or higher	345	72.9	13.1 (7.0)
Socioeconomic status	Low family SES	332	51.5	13.7 (6.7)
	Medium–high SES	313	48.5	12.1 (6.6)
ADHD diagnosis subtype	DSM-IV combined subtype	519	73.0	13.7 (6.7)
	DSM-IV predominantly inattentive subtype	43	6.0	9.9 (6.2)
	DSM-IV predominantly hyperactive-impulsive subtype	73	10.3	11.4 (6.9)
	DSM-III-R-only	76	10.7	11.8 (5.6)
ODD or CD diagnosis	DSM-IV ODD diagnosis	305	44.3	13.9 (6.7)
	DSM-IV CD diagnosis	131	19.0	15.1 (6.7)
	No CD or ODD diagnosis	252	36.6	11.0 (6.1)
Anxiety or depression diagnosis	Any DSM-IV anxiety diagnosis^a^	49	7.2	15.7 (7.7)
	Any DSM-IV depression diagnosis^a^	6	0.9	11.7 (6.0)
	No anxiety or depression diagnosis	630	92.2	12.8 (6.5)
Total sample		711	100	13.0 (6.6)

^a^Two children met criteria for a diagnosis of both anxiety and depression

Results of regression analyses of SCQ scores with clinical, cognitive and developmental outcomes are displayed in Tables 2 and 3.

**Table 2:** Association of the Social Communication Questionnaire with clinical outcomes: ADHD and comorbidities

Outcome variable	Unadjusted (max *N* = 711)			Adjusted^a^ (max *N* = 599)	Adjusted^b^ (max *N* = 599)
	B (Std. error)	OR (95 % CI)	*p* value*	*p* value*	*p* value*
DSM-IV combined ADHD diagnosis		1.06 (1.03–1.09)	1.6E−05	2.0E−03	N/A
ADHD symptoms: inattentive^c^	0.01 (0.00)		1.0E−03	7.1E−03	N/A
ADHD symptoms: hyperactive−impulsive^c^	0.01 (0.00)		1.1E−08	6.8E−06	N/A
ADHD symptoms: total	0.08 (0.01)		1.4E−08	5.9E−06	N/A
DSM-IV ODD diagnosis		1.03 (1.01–1.06)	4.2E−03	2.1E−03	0.013
DSM-IV CD diagnosis		1.06 (1.03–1.09)	1.1E−04	0.016	0.036
ODD symptoms	0.10 (0.01)		1.5E−14	2.6E−08	6.4E−06
CD symptoms^c^	0.02 (0.00)		2.3E−09	2.1E−05	3.9E−04
DSM-IV anxiety any diagnosis		1.06 (1.02–1.11)	3.9E−03	1.1E−03	2.5E−03
Anxiety symptoms^c^	0.02 (0.00)		7.9E-07	5.3E−06	1.2E−04
Depression symptoms^c^	0.01 (0.00)		1.4E−03	1.5E−03	0.022

*ADHD* attention deficit hyperactivity disorder, *ODD* oppositional defiant disorder, *CD* conduct disorder

* Statistical threshold to account for multiple testing: *p* value <0.003; all *p* values presented to two significant figures; direction of effect across analyses is consistent unless otherwise stated

^a^Adjusted for IQ, age, gender and family socio-economic status

^b^Adjusted for IQ, age, gender, family socio-economic status and ADHD severity

^c^Transformed

**Table 3:** Association of Social Communication Questionnaire with cognitive/developmental outcomes

Outcome variable	Unadjusted (max *N* = 711)			Adjusted^a^ (max *N* = 599)	Adjusted^b^ (max *N* = 599)
	B (Std. error)	OR (95 % CI)	*p* value*	*p* value*	*p* value*
WISC-IV full-scale IQ	−0.45 (0.08)		5.7E−08	1.1E−06^d^	1.6E−06^d^
WISC-IV Digit Span subtest	−0.07 (0.02)		8.6E−05	1.7E−04^d^	5.4E−04^d^
CANTAB IED completed stage 8		1.00 (0.97–1.04)	0.76	0.33	0.43
WORD Basic Reading	−0.05 (0.02)		0.020	0.99^e^	0.78^e^
WORD Spelling^c^	−4.47 (2.10)		0.034	0.95^e^	0.68^e^
WORD Reading Comprehension	−0.03 (0.02)		0.17	0.18^e^	0.13^e^
Motor problems		1.09 (1.06–1.12)	2.6E−09	5.2E−09	6.7E−07
Language problems		1.07 (1.04–1.10)	3.9E−06	7.8E−06	2.7E−03

*WISC-IV* Wechsler Intelligence Scale for Children version IV, *WORD* Wechsler Objective Reading Dimensions, *CANTAB IED* intra-extra dimensional shift task from the Cambridge Neuropsychological Battery

* Statistical threshold to account for multiple testing: *p* value <0.003; all *p* values presented to two significant figures; direction of effect across analyses is consistent unless otherwise stated

^a^Adjusted for IQ, age, gender and family socio-economic status

^b^Adjusted for IQ, age, gender, family socio-economic status and ADHD severity

^c^Transformed

^d^Not corrected for IQ

^e^Direction of effect opposite to that in the unadjusted analysis

### Clinical outcomes: ADHD and comorbidities

In terms of ADHD profile, higher SCQ scores were associated with presence of the ADHD combined subtype diagnosis in comparison to the other ADHD subtypes in the unadjusted model. SCQ scores were also positively associated with number of inattentive, hyperactive-impulsive symptoms and total number of ADHD symptoms. This pattern of results remained similar after adjusting for covariates.

Higher SCQ scores showed association with the presence of ODD and CD diagnoses and increasing ODD and CD symptoms. The pattern of results persisted after adjustment for covariates, though overall the strength of the associations decreased.

In the secondary test of CD symptoms, both aggressive (*B* = 2.66, *p* = 6.0 × 10^−5^) and covert (*B* = 1.79, *p* = 0.002) symptoms independently showed association with SCQ total. After adjusting for covariates, aggressive symptoms continued to show independent association with SCQ scores (*B* = 2.27, *p* = 0.002) but covert symptoms were no longer associated (*B* = 0.89, *p* = 0.16).

In the primary unadjusted analysis, SCQ scores were positively associated with presence of any anxiety diagnosis and increasing number of anxiety and depression symptoms. The pattern of results remained similar after adjustment for covariates, although the association with depression symptoms was less strong when ADHD severity was included.

### Cognitive/developmental outcomes

Full-scale IQ was found to be negatively associated with SCQ score. A secondary multivariate regression model was used to explore the relative contributions of the four indices of the WISC-IV to this association. There were no unique contributions of Verbal Comprehension Index, Perceptual Reasoning Index or Processing Speed (*p* > 0.05). However, there was a trend towards an association between higher SCQ scores and a lower Working Memory Index above and beyond contributions shared with the other three WISC-IV indices (unadjusted analysis: *B* = −0.07, *p* = 0.014; adjusted for all covariates: *B* = −0.09, *p* = 0.0029).

An analysis of the Digit Span subtest found that lower scores were significantly predicted by higher SCQ scores, a finding that persisted after adjusting for covariates. Detailed results can be found in Table 3.

In terms of EF, whether children successfully completed the extra-dimensional shift stage of the CANTAB IED task was not found to be associated with SCQ score. There was also no association of SCQ scores with WORD Basic Reading, Spelling Reading Comprehension after accounting for multiple testing, in any of the models.

Higher SCQ scores were associated with a greater likelihood of motor problems and general language problems.

### Secondary analysis of SCQ sub-domains

For each of the outcome variables showing a significant association with total SCQ score, additional tests were performed to determine whether any of the three SCQ sub-domains (social deficits, communication deficits and RRBs) showed an independent contribution to the observed association. After accounting for multiple testing, the sub-domains were generally not found to contribute independently to the associations seen with the outcome variables. The exceptions to this were that social deficits were independently associated with number of ODD symptoms (*B* = 0.12, *p* = 0.0002) and RRBs independently predicted hyperactive-impulsive symptoms (*B* = 0.03, *p* = 0.0005), anxiety symptoms (*B* = 0.05, *p* = 0.002) and presence of motor problems (OR = 1.25, 95 % CI = 1.12–1.40, *p* = 0.0001). These results are available from the authors upon request.

## Discussion

We set out to determine whether the presence of autistic traits in children with a diagnosis of ADHD indexes severity of phenotype in terms of clinical, cognitive and developmental features. The SCQ scores in our sample (mean = 13.0, SD = 6.6) are lower than reported in children ascertained primarily in terms of a diagnosis of ASD (mean = 22.3 [41]), as would be expected given that this sample consists of children with ADHD and those with a clinician’s diagnosis of ASD were not included. Furthermore, this mean is higher than previously reported in typically developing children (mean = 3.89, SD = 2.77 [31]; mean = 2.8, SD = 2.1 [32]) and somewhat higher than reported for other ADHD samples (mean = 8.5, SD = 6.2 [31]; mean = 11.6, SD = 5.5 [32]). Although not a direct comparison, the higher SCQ traits in our sample as compared with previous reported scores in controls are consistent with the many previous observations of high levels of comorbidity between ADHD and ASD at both diagnosis and trait level.

As predicted, the results on the whole suggest that autistic symptomatology, as indexed by total SCQ scores, within ADHD (even when known ASD cases are not included) is associated with a more severe phenotype in terms of several clinical, cognitive and developmental features. The results show that higher SCQ scores predict greater severity of ADHD symptoms and greater likelihood of combined subtype, more comorbid ODD, CD, anxiety and depression symptoms, lower cognitive ability (specifically, lower full-scale IQ and impairments in working memory as assessed by the Digit Span subtest) and a greater rate of general developmental (motor and language) problems. This pattern of results persisted even after taking into account potential confounding effects of age, IQ, gender and family SES and was found not to be driven by severity of ADHD itself. However, when covariates were included some of the associations weakened and *p* values fell below the threshold accounting for multiple testing, specifically in terms of depression symptoms and ODD and CD diagnoses. The most robust associations were found between SCQ score and ODD, CD and anxiety symptoms, working memory problems and motor problems, which persisted after accounting for ADHD symptoms. These results lend strength to the assertion that autistic symptoms independently drive the observed severity of these comorbid impairments, rather than simply being a proxy marker of ADHD symptom severity.

Although more working memory deficits were predicted by increasing SCQ score, there was no association detected between SCQ scores and whether children were able to successfully complete the extra-dimensional shift component of the IED CANTAB task, a further EF measure. Although previous studies have shown that unmedicated children with ADHD and children with ASD perform worse on this aspect of the IED task than controls [52–55], the current results suggest that autistic traits in ADHD do not index a greater deficit in set-shifting. A previous study utilising the IED task indicates that children with a joint diagnosis of ADHD and ASD may struggle more on this task than children with ASD without ADHD, but the study detected no differences in the performance of children with ADHD without ASD compared to the other groups, except that they took longer to complete the task [57]. EF is a broad construct and the current results suggest that different aspects of EF (e.g., working memory and set-shifting) need to be considered separately as they may show differential association with autistic traits.

Secondary analysis of the independent contributions of covert and aggressive CD symptoms to the association between SCQ scores and CD symptoms indicates that it is the aggressive symptoms which drive this association. This finding is in line with previous studies which have shown that CD traits in children with ADHD are a marker of greater clinical severity [58] and higher genetic risk [59], which appears to be driven by aggressive, rather than covert, CD symptoms [60, 61].

Secondary analyses were also performed to determine the relative contributions of the three SCQ sub-domains (social deficits, communication deficits and RRBs) to observed main associations. Results indicated that for most outcomes associated with SCQ total score, the three sub-domains did not show any independent association above and beyond the variance they explained in common, although with a few exceptions. After correction for multiple testing, associations were observed between the sub-domain of social deficits and number of ODD symptoms, as well as the sub-domain of RRBs and hyperactive-impulsive symptoms, anxiety symptoms and general motor problems. The results suggest that RRBs in children with ADHD, as assessed by the SCQ, may index a more general dysfunction of motor processes (i.e., hyperactivity, impulsivity and general motor problems in the form of late onset walking and clumsiness). Previous research has shown that all three sub-domains contribute to the higher SCQ scores in children with ADHD when compared to their unaffected siblings and typically developing controls [31], although a smaller study found this was the case with social and communication problems but not repetitive behaviours compared with typically developing controls [32]. The current study addresses the proposed independence [34] of these sub-domains and suggests that the variance that is common across them is broadly relevant to the clinical, cognitive and developmental outcomes assessed, with only a handful of unique effects.

An alternative approach to investigate the effects of comorbidity between ASD and ADHD is to assess whether a sample of children ascertained primarily in terms of a diagnosis of ASD shows similarly elevated comorbid and cognitive problems. Studies generally find that children with ADHD + ASD have elevated ASD symptomatology, higher rates of comorbid ODD, CD, anxiety and depression problems and lower IQ scores than children with ASD-only [62–66] although others do not [28, 67]. Thus it would seem that, in general, ADHD problems in children with ASD also index more comorbid and cognitive problems, lending support to the removal of the dual diagnosis exclusion proposed for DSM-5 [27].

The current study includes the following strengths but also has a number of limitations. The sample size was large and rigorously phenotyped for ADHD and comorbid conditions, using the CAPA for every participant, and a comprehensive assessment of cognition was undertaken. This study assessed ADHD using DSM-IV criteria that require excluding cases of ASD and, therefore, children with comorbid ASD were not included. However, ASD scores were not assessed by standardised interview and observations. Given that children with ADHD and ASD show higher rates of ID, children were not excluded on the basis of IQ so as not to lose clinically valuable information. Likely for historical reasons [68], children with ID have generally been excluded from ADHD research studies but not from ASD research. The pattern of our results remained the same when ID cases (*N* = 85) were excluded (results available from first authors).

It is not clear whether the SCQ has validity for autistic traits in ADHD in the same way that it does in those with ASD and the general population, although that could easily also apply to assessment of other forms of comorbid psychopathology in ADHD (e.g., CD). For instance, the item on “complicated movements of the body” (part of the sub-domain of RRBs) may be interpreted as referring to straightforward hyperactivity, an idea that has been proposed by others in general with regard to parental reports of children’s behaviour [32, 69]. An analysis of the properties of the SCQ in a sample of young people ‘at risk’ for ASD found that 25 % of those who screened false-positive for ASD went on to receive a diagnosis of ADHD or hyperkinetic disorder [70], but to date it has not been examined in detail as to whether the SCQ has good discriminant validity of autistic symptoms in a population with ADHD specifically.

Although the continuous analysis of SCQ scores can be viewed as a strength, it is possible that some of the children with particularly high SCQ scores in this sample would have a clinical diagnosis of an ASD, were the diagnosis to be revisited and assessment with the ADI-R [43] or the Autism Diagnostic Observation Schedule (ADOS) [71] carried out; other studies of ASD traits in ADHD have excluded children over a certain threshold of ASD problems who are then confirmed as having ASD on subsequent assessment, using the Parental Account of Children’s Symptoms [31]. It is worth noting that analysing the data using the SCQ as a binary variable (dividing the children into groups based on the screening cut-off threshold used in the literature [41]) and thus comparing children with total SCQ <15 (*N* = 433) to those with SCQ score ≥15 (*N* = 278) gives the same pattern of results and direction of effects as reported for the continuous analysis (results available from the first authors). Given that a previous study suggested that a cut-off point of 11 is particularly meaningful in identifying children with ADHD who may have ASD [30], it is of interest to note that 61.2 % (*N* = 435) of the children in this sample have SCQ scores ≥11.

There was a wide age range across the sample and as such, it is important to bear in mind that ADHD subtypes are not stable over time and their validity as categorical entities is questionable although symptom domains as dimensions seem to be better validated [72]. Indeed, longitudinal developmental trajectories of hyperactive-inattentive traits in the general population are diverse [73]. This issue was partially addressed by excluding those with remitted ADHD, and using symptom count measures as well as categorical subtype diagnoses.

A further limitation of the study is that although parents were asked to withhold stimulant medication for 24 h prior to cognitive testing, a small proportion of parents (14.4 %) did not follow these instructions. However, this information could not be reliably used as a covariate when examining the association of SCQ score with the cognitive tests due to the high variability of the types and dosages of medication the children took and perhaps more importantly, the variability in time of day the assessments took place (ranging from morning to evening).

Although no associations were found between reading and spelling and SCQ scores, the WORD was only assessed in children between 5 and 11 years old (*N* = 375). Similarly, the CANTAB IED task was only available for a proportion of the children (*N* = 252), which may have reduced the power to detect potential effects. The measures of general motor and language development were broad screening questions indicative of developmental problems rather than detailed validated measures, which precluded the assessment of more subtle associations of autistic symptomatology with general development.

Finally, a major limitation of this cross-sectional data is that without longitudinal follow-up it is not possible to ascribe a direction of effect to the observed associations between SCQ scores and the clinical and cognitive outcomes. Related to this point, in the secondary analyses of covert and aggressive CD symptoms, as well as that for the indices of the WISC-IV IQ, the analytical approach differed from the primary analysis (i.e., in the CD/IQ secondary analyses, SCQ score was the outcome not the predictor) to be able to assess the unique contribution of each of the CD/IQ components to the observed main associations using multivariate regression. Given that we are unable to ascribe a direction of effect to the observed associations in view of the cross-sectional nature of the data, this analytical strategy provides an insight into the origin of effects.

### Clinical implications and suggestions for future research directions

Overall, the findings suggest that in children with ADHD, the presence of autistic symptomatology (that is not at a diagnostic level) indexes a more impaired phenotype—encompassing not only severity of ADHD, but also comorbid psychopathology, general cognitive and working memory deficits, and motor and language developmental problems. These findings corroborate and extend previous research [29–31] and would benefit from subsequent replication with other large samples. Ideally, a set of controls also assessed on all measures would be included, to determine the true independent and joint relationships of ADHD and ASD traits with other forms of psychopathology in children.

With the current proposed changes to DSM-5 and ICD-11, clinical thinking and practice are already moving beyond the restriction of precluding a dual diagnosis of ADHD and ASD. Our results further suggest the merit of this. They highlight that rather than attempting to generate mutually distinctive diagnostic classes, it would be worthwhile for clinicians to acknowledge the strong overlap in young people with ADHD and to consider levels of socio-communicative and RRB traits in those who do not meet diagnostic criteria for ASD, as they index higher levels of comorbidity and cognitive and developmental problems.

The presence of undiagnosed deficits in social and communication abilities may also have implications for the effectiveness of behavioural strategies and pharmacological treatment aimed to ameliorate ADHD symptoms. Whilst there is evidence for potential beneficial effects of stimulant medication on ADHD traits in children with ASD [74], (although the evidence for the effectiveness of atomoxetine in reducing ADHD behaviours in ASD is more mixed [75, 76]), it is not yet clear as to how the presence of ASD symptoms in children with ADHD may impact on response to ADHD medication. Nevertheless, if strategies aimed at addressing autistic symptomatology in ADHD could be implemented, these might have the potential to improve the effect of both behavioural and pharmacological ADHD interventions, reduce comorbidities and their associated distress and facilitate learning.

## Electronic supplementary material

Below is the link to the electronic supplementary material.

Supplementary material 1 (DOCX 11 kb)
