# Supplementary material for: Autistic traits in children with ADHD index clinical and cognitive problems
Source: Eur Child Adolesc Psychiatry. 2013 Apr 25;23(1):23–34. doi: 10.1007/s00787-013-0398-6 (PMC3899449; doi:10.1007/s00787-013-0398-6)
Supplement: Supplementary file 1 — Supplementary material 1 (DOCX 11 kb) [file 787_2013_398_MOESM1_ESM.docx]

**Supplementary Table 1 –** Social Communication Questionnaire item sub-domain classification

| **SCQ item** | **DSM-IV Sub-domain** |
| --- | --- |
| 2 | Communication |
| 3 | Communication |
| 4 | Communication |
| 5 | Social |
| 6 | Communication |
| 7 | Communication |
| 8 | Communication |
| 9 | Repetitive |
| 10 | Social |
| 11 | Social |
| 12 | Repetitive |
| 13 | Repetitive |
| 14 | Repetitive |
| 15 | Repetitive |
| 16 | Repetitive |
| 17 | Repetitive |
| 18 | n/a |
| 19 | Repetitive |
| 20 | Social |
| 21 | Communication |
| 22 | Social |
| 23 | Social |
| 24 | Social |
| 25 | Social |
| 26 | Social |
| 27 | Social |
| 28 | Social |
| 29 | Social |
| 30 | Social |
| 31 | Social |
| 32 | Social |
| 33 | Social |
| 34 | Communication |
| 35 | Communication |
| 36 | Social |
| 37 | Social |
| 38 | Social |
| 39 | Communication |
| 40 | Social |
